# 25 de Abril Sempre! Portuguese Science and the 50th anniversary of the Carnation Revolution

**DOI:** 10.1038/s42003-024-06229-y

**Published:** 2024-05-07

**Authors:** Tiago Dantas, Carla M. C. Abreu, Maria J. G. De-Castro, Ana Rita Grosso, Joao de Sousa Valente, Gabriela da Silva Xavier

**Affiliations:** 1 i3S—Instituto de Investigação e Inovação em Saúde, Universidade do Porto, Porto, Portugal; IBMC—Instituto de Biologia Molecular e Celular, Universidade do Porto, Porto, Portugal; 2 Associate Laboratory i4HB—Institute for Health and Bioeconomy, NOVA School of Science and Technology, Universidade NOVA de Lisboa, 2829-516 Caparica, Portugal; 3 UCIBIO—Applied Molecular Biosciences Unit, Department of Life Sciences, NOVA School of Science and Technology, Universidade NOVA de Lisboa, 2829-516 Caparica, Portugal; 4 Communications Biology, Madrid, Spain; 5 Institute of Metabolism and Systems Research, University of Birmingham, Birmingham, UK

**Figure:**
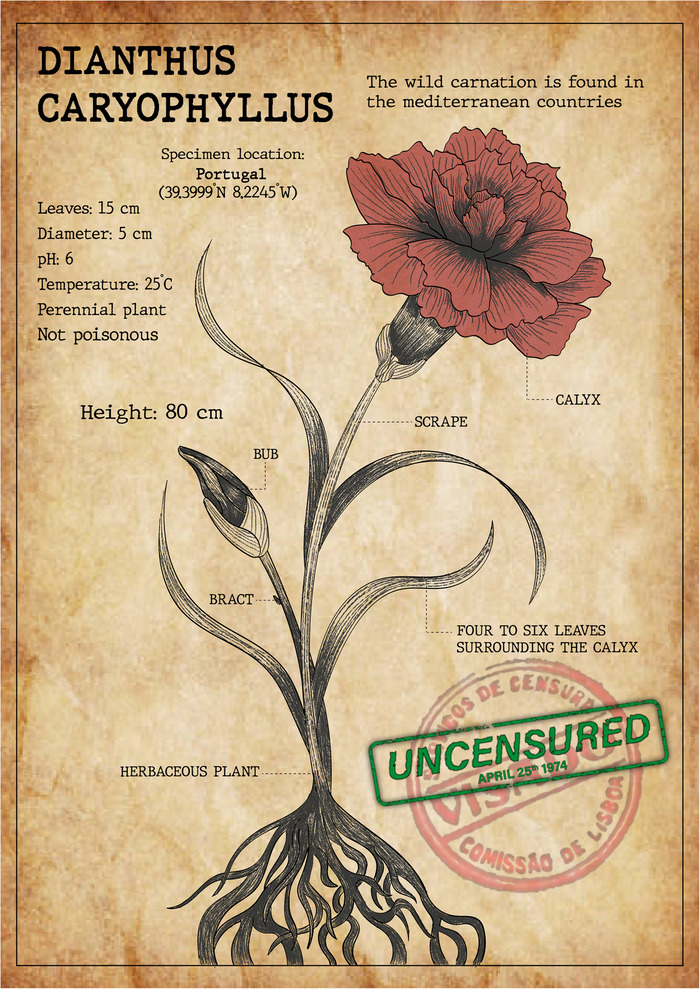
*Artistic illustration alluding to the Carnation Revolution that liberated Portugal from the high levels of censorship that had been imposed by a dictator’s regime*. *Maria J. G. De-Castro*.

**In 2024, Portugal celebrates the 50th anniversary of the Carnation Revolution, which brought down a long dictatorship and re-instated elemental civil liberties and democracy in the country. For Portuguese science, this revolution meant a democratisation of access to the scientific career and an increased investment in scientific research, which culminated in an unprecedented rise in scientific output.** ***Communications Biology*** **joins this anniversary and celebrations of freedom and democracy as basic pillars of scientific endeavour.**

The period during the world wars saw a rise in nationalist and autocratic ideologies across Europe. In Portugal, the 1920s was a decade marked by political instability and economic stagnation, setting the stage for a pivotal political turn: a military *coup d’ État* that ultimately established a 48-year-long dictatorship led by António de Oliveira Salazar and his successor Marcelo Caetano. The regime’s isolationist policies heavily restricted fundamental civil liberties and rights: there was no freedom of the press or association, limited access to education overall precarious life conditions and limited access to basic resources, in turn resulting in a low life expectancy and high number of childbirth deaths. The limitation of the basic rights was strongly reflected on the woman’s condition; they were, for the most, excluded from learning and were not permitted to vote or travel without consent from parents or husbands. As for the Portuguese scientific system during the regime, it had limited dimension and diversity, and was isolated from the scientific community at large. While universities were a rare place of critical thinking and resistance against the regime, the systemic impeded any margin for dissidence. The penalty would be expulsion, exile, or imprisonment. Scientific funding was residual, state-distributed and primarily focused on areas of immediate political interest.

In 1974, the Portuguese political regime reached a tipping point. The endless war against the self-determination of the Portuguese-speaking African countries was hopeless and led the country to international isolation. The nearly 50 years of dictatorship left the Portuguese civil society stagnant and far behind the rest of Europe. On the 25th of April 1974, a movement of the armed forces, supported by civil society, peacefully marched into Lisbon carrying carnations in their guns and seized power. What emerged was a young democracy willing to restore basic civil rights and adopt key health and education policies. Today’s Portugal has literacy and life expectancy indicators on par with the rest of Europe, with lower rates of early leavers from education and training and higher tertiary educational attainment than the EU-27 average, as shown in figures from the European Commission^1^. However, the data indicate that there are persistent regional differences, with a lower than EU-27 average rate in the Central region vs. a higher than EU-27 average rate in the autonomous region of the Azores. Levelling such disparities would further democratise education and increase overall attainment, which is achievable with the increase in overall spending on education and measures to support access to and enrolment in higher education.

Importantly, the Carnation Revolution also provided fertile ground for Portuguese science to flourish. The push for universal education allowed the country to tap into its human talent, by democratising access to the scientific career to all, specifically to people from less privileged socioeconomic backgrounds and women. Women were barely represented in Portuguese PhD graduates in 1974, but make up more than half of them today^2^. Moreover, alignment with European values allowed Portugal to join the European Union in 1986, yielding two major positive effects: (1) free movement across borders meant an opportunity for the internationalisation of researchers, fostering knowledge exchange and collaboration; and (2) access to European funds allowed for crucial investment in personnel, research and development (R&D) projects, and infrastructure. In parallel, state investment grew 17 times from 0.1% to 1.7% of gross domestic product (GDP) by 2022^3^. Against this backdrop, science in Portugal experienced explosive growth, marked by a boom in the number of universities and research institutes, and a 25-fold increase in R&D employment. However, the most remarkable metric is scientific output, as evidenced by a staggering increase in publication quality and volume, largely surpassing the expectations considering the nation’s investment made in funding and personnel^4^. Increasing efforts for science dissemination to the public, e.g., through national agencies such as Ciệncia Viva, increases the visibility and role of science in everyday life to the public and would serve to increase engagement from the wider population.

“The push for universal education allowed the country to tap into its human talent, by democratising access to the scientific career to all, specifically to people from less privileged socioeconomic backgrounds and women.”

The Portuguese scientific panorama of today is one of the greatest achievements of the nation’s post-Carnation revolution era. Nevertheless, the gap with the rest of Europe is not yet closed. In many metrics, and despite the significant progress, Portugal lags behind in terms of scientific investment (as measured in percentage of GDP) and resources, both material and human. Other diagnosed insufficiencies are the limited articulation with the private sector and the limited incentive to foster novel and diverse collaborations which undermines meritocracy. Yet, today’s Portuguese scientific community includes a vibrant and highly qualified generation of young scientists—both in Portugal and abroad—that was educated on the “values of April” and that are ready to take leadership in upcoming challenges and drive scientific research to new heights.
